# Impact of maternal first trimester treatment regimen on the outcome of valproate exposed pregnancies: an observational Embryotox cohort study

**DOI:** 10.1038/s41598-023-50669-1

**Published:** 2024-01-05

**Authors:** Anne-Katrin Fietz, Marlies Onken, Stephanie Padberg, Christof Schaefer, Katarina Dathe

**Affiliations:** 1 Charité – Universitätsmedizin Berlin, Corporate Member of Freie Universität Berlin and Humboldt-Universität zu Berlin, Institute of Clinical Pharmacology and Toxicology, Embryotox Center of Clinical Teratology and Drug Safety in Pregnancy, Augustenburger Platz 1, 13353 Berlin, Germany; 2 Charité - Universitätsmedizin Berlin, Corporate Member of Freie Universität Berlin and Humboldt-Universität zu Berlin, Institute of Biometry and Clinical Epidemiology, Charitéplatz 1, Berlin, Germany

**Keywords:** Drug safety, Pharmacology, Epilepsy, Outcomes research, Neurological disorders

## Abstract

Effects of valproate (VPA) dose and treatment discontinuation during the first trimester of pregnancy on the risks of spontaneous abortions (SAB) and major birth defects were analyzed. Pregnancies with first trimester VPA exposure (n = 484) prospectively recorded by the German Embryotox center in 1997–2016 were compared with a randomly selected, non-exposed cohort (n = 1446). The SAB risk was not significantly increased in the VPA cohort [HR_adj_ 1.31 (95% CI 0.85–2.02)] but major birth defects were significantly more frequent [8.7% vs. 3.4%; OR_adj_ 2.61 (95% CI 1.51–4.50)]. Risk was even higher in pregnancies with no VPA discontinuation in first trimester [OR_adj_ 3.66 (95% CI 2.04–6.54)]. Significant ORs were found for nervous system defects in general [OR_adj_ 5.69 (95% CI 1.73–18.78)], severe microcephaly [OR_adj_ 6.65 (95% CI 1.17–37.68)], hypospadias [OR_adj_ 19.49 (95% CI 1.80–211)] and urinary system defects [OR_adj_ 6.51 (95% CI 1.48–28.67)]. VPA dose had a stronger effect than antiepileptic poly- versus monotherapy; for VPA dose ≥ 1500 mg/day the OR_adj_ was 5.41 (95% CI 2.32–12.66)]. A daily dose increase of 100 mg was calculated to raise the risk for major birth defects by 15% [OR 1.15 (95% CI 1.08–1.23)]. Overall, maternal first trimester treatment regimen had a relevant impact on birth defect risk.

## Introduction

The teratogenicity of valproate (VPA) is well-established, with evidence having accumulated since the 1980’s^1–10^. Specific birth defects like neural tube defects^2,6,10,11^, cardiac septal defects^2,6,9,10^, oro-facial clefts^2,6,10^, hypospadias^2,6,9,10^ and skeletal and limb malformations^6,10^ have been associated with VPA. Previous research revealed dose-dependency of VPA’s teratogenicity^3–6,8,9,12^. However, applied dose categories were heterogeneous and study results difficult to compare. Antiepileptic (AED) polytherapy including VPA was discussed to carry a higher risk than VPA monotherapy^3,12–15^. Moreover, neurodevelopmental disorders in childhood have been associated with VPA exposure in pregnancy^16,17^. Interestingly, data regarding the risk of spontaneous abortions (SAB) and VPA exposure during pregnancy are scarce and conflicting^1,9,18–22^.

The European Commission Pharmacovigilance Risk Assessment Committee (PRAC) of the European Medicines Agency (EMA) issued new warnings and restrictions of VPA use in 2014^23^ with further tightening in 2018^24^, aiming at avoidance of VPA for girls and women of childbearing potential. Following these restrictions, VPA should only be considered in women of childbearing age when safer alternatives are ineffective such as in some types of generalized epilepsy^25^. Ethical as well as risk–benefit aspects of VPA treatment for mother and unborn child have been widely discussed^26–33^. Several studies showed increased risks of unsatisfactory seizure control after withdrawal of VPA or switch to another AED during pregnancy^26,34^, or in women of childbearing potential in general^27–30^. In contrast, there are no studies on the time-dependent effects of VPA discontinuation during the first trimester on the risks of birth defects and pregnancy loss. So far, there has been only one study by Vajda et al.^26^, which analyzed the risk of major birth defects after pre-conception withdrawal of VPA treatment.

It is important to address changes in organ-specific susceptibility to toxic agents when analyzing drug exposure effects on pregnancy outcomes. Exposure to harmful substances can lead to negative effects on the embryo or fetus throughout pregnancy. The period most sensitive to teratogens is the first trimester, where the defined development of anatomical structures and organs mainly takes place and toxic exposure may result in major birth defects or spontaneous abortion. We analyzed the German Embryotox database focusing on the impact of VPA dose, treatment discontinuation, and mono- versus polytherapy within the first trimester on the risk of major birth defects and SAB.

## Methods

The Embryotox Center of Clinical Teratology and Drug Safety in Pregnancy (Embryotox) offers consultation on drug safety in pregnancy for health care professionals (HCP) and patients, with about 15,000 requests per year. Upon contact to Embryotox, details on all drug exposures (duration of treatment, dose, ATC-codes), treatment indications (MedDRA) and maternal medical history are recorded. Approximately eight weeks after the expected date of delivery, follow-up data are obtained by a standardized procedure using a questionnaire with a response rate of approximately 75%. Details on further drug exposures, course of pregnancy and delivery, and neonatal outcome including congenital anomalies are asked for. Data are generated iteratively from first contact in pregnancy to follow-up. In cases of incomplete or inconsistent response, particularly with respect to details of drug exposure and pregnancy outcome, the patient and/or her HCP are contacted for further information and medical records. Following this case by case plausibility check, data are archived (Embryotox database VigilanceOne, PharmApp Solutions GmbH). In addition, Embryotox serves as a national clearinghouse for suspected adverse drug reactions in pregnancy. A description of Embryotox procedures can be found in Dathe et al.^35^.

### Study design

This observational cohort study is based on prospectively ascertained pregnancies exposed to VPA at least during first trimester (independent of treatment duration) and with complete follow-up. Our study follows the recommendation of the Strengthening the Reporting of Observational studies in Epidemiology (STROBE) statement^36,37^. Pregnancies enrolled between January 1997 and December 2016 and exposed to VPA were compared to a randomly selected cohort unexposed to known teratogens or fetotoxicants at any stage of pregnancy, matched at a ratio 3:1 by year of enrollment. Definition of exclusion criteria, exposure, pregnancy outcomes and mono- and polytherapy are summarized in Table S1. Assignment to major birth defects was performed according to EUROCAT^38^ independently by two experienced clinicians blinded to exposure status.

Approval was obtained from the ethics committee of the Charité—Universitätsmedizin Berlin (EA2/130/18). The study was registered at the German Clinical Trial register (DRKS00015636, date of registration: 23.10.2018) and listed at the WHO International Clinical Trials Registry Platform. The procedures used in this study adhere to the tenets of the Declaration of Helsinki.

### Statistical analysis

Crude rates for SAB, elective termination of pregnancy (ETOP), stillbirth and live birth can be biased due to delayed study entry and competing risks^39^. Instead, cumulative incidences (CIF) were calculated using event history analysis for cause-specific sub-distributions of competing risks incorporating left truncation^39,40^. The effect of VPA exposure was estimated using a Cox proportional hazards regression model. The time-dynamic pattern of VPA exposure was accounted for using multistate models^41^. The multistate model by Bluhmki et al.^42^ was used, which classifies women for each point in time during pregnancy into the states “exposed” or “non-exposed (discontinued)” to VPA. Landmarking^43^ for probability estimation of pregnancy outcomes was then applied. The VPA cohort was stratified by discontinuation time into the groups “early discontinuation” (≤ GW 5 + 0), “moderate discontinuation” (between GW 5 + 1 and GW 7 + 0) and “late discontinuation” (at GW 7 + 1 or later)^42,44^. To avoid conditioning on the future^45^, the analysis was conditioned on observed and event free women at GW 7 + 1, which corresponds to the lower limit for late VPA discontinuers. For each discontinuation group, the probability for pregnancy outcomes was calculated. The effect of discontinuation on the hazard of SAB was estimated by applying two different Cox proportional hazards models^42^. First, the impact of time-dependent discontinuation on SAB risk was estimated by comparing the relative change in hazard of SAB between women having discontinued VPA in the first trimester and women remaining exposed to VPA throughout the first trimester. Second, time-specific discontinuation was estimated in the subgroup of all women having discontinued VPA in the first trimester and measures the relative change in the hazard of SAB for discontinuing VPA one week later.

Crude rates of major birth defects excluding genetic disorders were calculated by the number of live-born infants and fetuses affected with major birth defects divided by all live-born infants plus pregnancy losses with major birth defects. Effect estimation for major birth defects regarding VPA treatment, discontinuation groups (early discontinuation ≤ GW 5 + 0, moderate discontinuation GW 5 + 1 to 7 + 0, late discontinuation GW 7 + 1 to 12 + 6 and no discontinuation in first trimester ≥ GW 13 + 0), maximum dose in first trimester, mono- and polytherapy, and for preterm birth was based on odds ratios (OR) using logistic regression. ORs for affected EUROCAT organ systems and subgroups of congenital anomalies^38^ were calculated if there were more than three events per category. Extreme values of maximum doses due to suicide attempts (30,000 mg/day, 18,000 mg/day and 9000 mg/day) were excluded from descriptive statistics and logistic regression models. Dose category groups based on the maximum dose in first trimester were chosen according to Tomson et al.^12^ (< 700 mg/day, 700 to < 1500 mg/day, ≥ 1500 mg/day).

Birth weight and head circumference were evaluated using the percentile values derived from the German perinatal survey^46,47^. The effect of VPA was assessed using the standard deviation score (SDS) in a linear regression model. All regression models are presented with 95% confidence intervals (CI).

To reduce possible confounding, all regression models were adjusted for the same set of relevant baseline covariates (labeled in Table S2) by either incorporating the covariates directly in the regression models or by using a stratified analysis based on the quintiles of the propensity score (PS)^48^. Imputation of missing values and estimation of the PS was performed based on standard procedures by Embryotox^44^. R version 4.0.2 (R Core Team) was used.

### Consent to participate

Informed consent was obtained from all individual participants included in the study.

## Results

During the study period 1997–2016, n = 484 VPA exposed pregnancies met the study criteria. These were compared with n = 1446 prospectively ascertained pregnancies not exposed to VPA or other teratogens (Fig. S1).

### Maternal characteristics

Maternal characteristics differed significantly between the VPA and comparison cohort (Table S2). Women in the VPA cohort were younger (29 years vs. 32 years), had a higher BMI (24.4 vs. 22.8), a lower level of advanced education (28.2% vs. 62.1%) and smoked more (26.3% vs. 12.1%). They had less frequently planned their pregnancy (78.4% vs. 90.7%), had a higher rate of previous ETOP (12.7% vs. 6.8%) and more often previous children with major birth defects (2.7% vs. 0.7%). In addition, they started folic acid intake less often preconception (24.6% vs. 42.1%) and the first contact to Embryotox was earlier (GW 7 + 6 vs. GW 8 + 6).

### Treatment indications and exposure to VPA

Epilepsy was the most frequent treatment indication for VPA (69%), followed by bipolar disorder (11%), and other psychiatric disorders or migraine; further information on treatment indication is listed in Table S3. Discontinuation status in first trimester was known for the majority of women (89%, n = 430/484). Almost all women were already on VPA exposure at the beginning of pregnancy (96%, n = 467/484) and the majority of women took valproate on a regular basis (98%, n = 476/484). Nearly half of the women (42%, n = 179/430) discontinued VPA in the first trimester (median GW 5 + 6, IQR GW 4 + 6 − 7 + 0), and additional 22 women discontinued after the first trimester (Fig. S2).

VPA treatment during first trimester was monotherapy in 68% (n = 292/428) and polytherapy in 32% (n = 136/428). For the remaining n = 56 pregnancies, assignment to mono- or polytherapy was inconclusive. Lamotrigine was the most frequent substance used in a polytherapy regimen (47%, n = 64), followed by levetiracetam (16%, n = 22) and carbamazepine (13%, n = 18). Additional substances of polytherapy are summarized in Table S4. In contrast to other treatment indications, women with epilepsy were almost six times longer exposed during pregnancy (median duration 250 days), less often discontinued VPA in first trimester (23% vs. 60–94%) and used VPA more often in antiepileptic polytherapy regimen (41% vs. 10–14%) (Table S3).

### Pregnancy outcome

**Figure 1:**
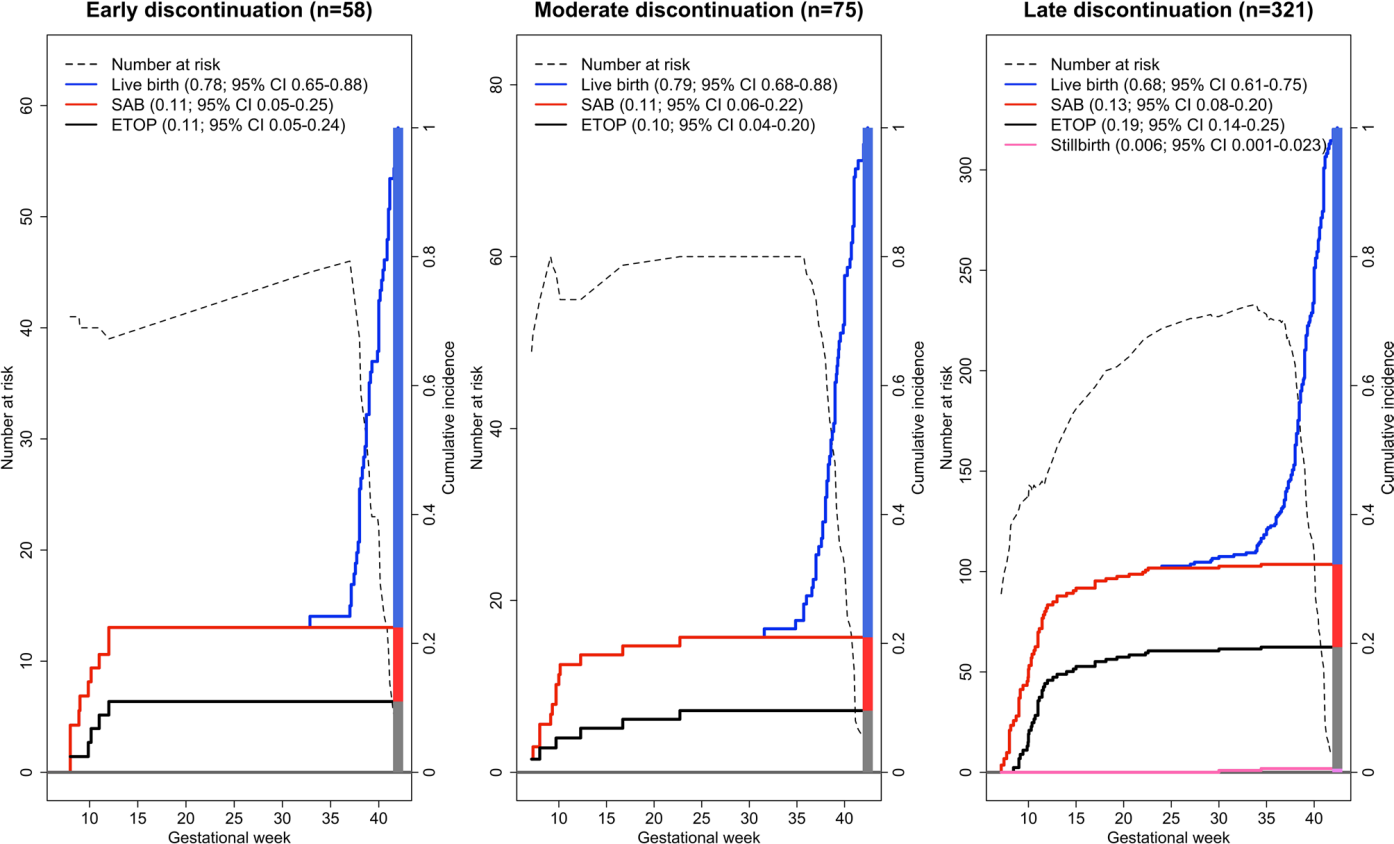
Stacked cumulative incidences for the pregnancy outcomes live birth, spontaneous abortion (SAB), elective termination of pregnancy (ETOP) and stillbirth stratified by early (≤ gestational week (GW) 5 + 0), moderate (GW 5 + 1 ≤ GW 7 + 0) and late (still exposed at GW 7 + 1) treatment discontinuation with the number of pregnancies at risk for each gestational day (dotted line). For multiple pregnancies with identical outcomes, only one outcome was considered. Pregnancies with incomplete information of discontinuation (seven live births, seven SAB, 13 ETOP) and pregnancies ending before GW 7 + 1 (three SAB) were excluded.

Pregnancy outcomes are summarized in Table 1. The risk for SAB was slightly but not significantly increased after VPA exposure (Table 1, Fig. S3). Cumulative incidences for SAB were independent of the time of VPA discontinuation (early < GW 5 + 0; moderate GW 5 + 1 to 7 + 0; late ≥ GW 7 + 1) (Fig. 1). The results are in line with the Cox proportional hazards models including discontinuation as a time-varying covariate. The risk of SAB was not increased for continued exposure to VPA in first trimester in contrast to discontinuation [HR 0.71 (95% CI 0.35–1.46)], and was not increased by later discontinuation time in the subset of women with first trimester discontinuation [HR 1.07 (95% CI 0.79–1.45)].

**Table 1:** Pregnancy outcomes of the valproate and comparison cohort.

Pregnancy outcomes	VPA n (CIF in %)	Comparison n (CIF in %)	HR (95% CI)	HR_adj_* (95% CI)
Pregnancies	484	1446^1^		
Live birth	382 (62.0)	1277 (76.6)	0.98 (0.87–1.10)	0.96 (0.85–1.09)
SAB	40 (18.8)	99 (15.7)	1.18 (0.82–1.71)	1.31 (0.85–2.02)
ETOP	60 (18.8)	66 (7.4)	2.82 (1.98–4.00)	2.26 (1.49–3.41)
Stillbirth	2 (0.4)	5 (0.3)		
Liveborn infants	385	1299		
Stillborn infants	2	7		

*CI* confidence interval, *CIF* cumulative incidence function, *ETOP* elective termination of pregnancy, *HR* hazard ratio, *n* number of pregnancies/infants, *SAB* spontaneous abortion, *VPA* valproate.

^1^Including one pregnancy of twins resulting in one liveborn infant and one SAB and one pregnancy of triplets resulting in three stillborn infants.

*Adjusted by using the quintiles of the propensity score incorporating maternal age, use of nicotine and alcohol, number of previous deliveries, number of previous spontaneous abortions, pregestational diabetes, folic acid intake preconception.

The rate for ETOP was significantly higher after VPA exposure (Table 1, Fig. S3) and there was a slight increase for ETOP with longer duration of VPA treatment (Fig. 1).

#### Neonatal characteristics

Neonatal parameters are summarized in Table S5. The rate of preterm birth was higher in the VPA cohort but not statistically significant after adjustment. Birth weight and head circumference were lower in the VPA cohort but adjusted SDS differences were not statistically significant.

#### Birth defects

Major birth defects, affected organ systems and subgroups are shown in Table 2. Overall, the risk of major birth defects after VPA exposure was significantly increased and in particular for defects within the group nervous system in general, severe microcephaly, urinary system defects and genital defects (driven by a statistically significant increase in the risk of hypospadias in male infants). The risks for spina bifida, cardiac defects in general, atrial septal defects, oro-facial clefts, limb disorders overall, limb reduction defects, polydactyly and the group of other anomalies/syndromes were increased, but statistical significance was not reached. Details of VPA exposed pregnancies with major birth defects are summarized in Table S6.

**Table 2:** Crude rates and results of logistic regression for major birth defects, corresponding affected organ systems and subgroups defined by EUROCAT^38^.

	VPA n (%)	Comparison n (%)	OR (95% CI)	OR_adj_* (95% CI)
Major birth defect	34/393^1^ (8.7)	44/1306^2^ (3.4)	2.72 (1.71–4.31)	2.61 (1.51–4.50)
Nervous system	11/389 (2.8)	6/1302 (0.5)	6.29 (2.31–17.11)	5.69 (1.73–18.78)
Spina bifida	4/389 (1.0)	1/1300 (0.1)	13.50 (1.50–121.10)	11.00 (0.84–143.91)
Severe microcephaly^3^	5/385 (1.3)	3/1299 (0.2)	5.68 (1.35–23.89)	6.65 (1.17–37.68)
Ear, face and neck	1/385 (0.3)	1/1299 (0.1)		
Congenital heart defects^4^	9/388 (2.3)	18/1301 (1.4)	1.69 (0.75–3.80)	1.50 (0.59–3.85)
Transposition of great vessels	1/386 (0.3)	1/1299 (0.1)		
Ventricular septal defect (VSD)	4/386 (1.0)	12/1299 (0.9)	1.12 (0.36–3.50)	0.97 (0.26–3.58)
Atrial septal defect (ASD)	2/386 (0.5)	3/1299 (0.2)	2.25 (0.37–13.51)	2.91 (0.35–24.04)
Pulmonary valve stenosis	1/386 (0.3)	2/1299 (0.2)		
Coarctation of aorta	1/385 (0.3)	−		
Respiratory system	1/386 (0.3)	2/1299 (0.2)		
Oro-facial clefts	5/387 (1.3)	3/1300 (0.2)	5.66 (1.35–23.78)	3.04 (0.61–15.23)
Cleft lip with palate	2/386 (0.5)	1/1299 (0.1)		
Cleft palate	3/386 (0.8)	2/1300 (0.2)	5.08 (0.85–30.53)	3.47 (0.47–25.86)
Digestive system	2/368 (0.5)	1/1299 (0.1)		
Ano-rectal atresia and stenosis	1/386 (0.3)	−		
Urinary system	6/386 (1.6)	4/1299 (0.3)	5.11 (1.44–18.21)	6.51 (1.48–28.67)
Multicystic renal dysplasia	1/385 (0.3)	–		
Genital tract	7/386 (1.8)	1/1299 (0.1)	23.97 (2.94–195.31)	21.85 (1.97–242.86)
Hypospadias (male)	7/214 (3.3)	1/664 (0.2)	22.42 (2.74–183.29)	19.49 (1.80–210.73)
Limb disorders	5/387 (1.3)	7/1300 (0.5)	2.42 (0.76–7.66)	2.38 (0.61–9.24)
Limb reduction defects	2/387 (0.5)	2/1299 (0.2)	3.37 (0.47–23.99)	2.28 (0.21–24.30)
Polydactyly	2/385 (0.5)	2/1299 (0.2)	3.39 (0.48–24.12)	3.17 (0.38–26.79)
Syndactyly	1/385 (0.3)	1/1299 (0.1)		
Other anomalies/syndromes	2/385 (0.5)	3/1301 (0.2)	2.26 (0.38–13.57)	3.57 (0.42–30.47)
Craniosynostosis	1/385 (0.3)	1/1299 (0.1)		

Classification of major birth defects according to the EUROCAT-guideline^38^. Multiple affected organ systems in one infant or fetus are counted separately.

*CI* confidence interval, *ETOP* elective termination of pregnancy, *n* number of liveborn infants plus fetuses affected with major birth defects, *OR* odds ratio, *SAB* spontaneous abortion, *VPA* valproate.

^1^Including one SAB, six ETOP and one stillbirth.

^2^Including one SAB, five ETOP and one stillbirth.

^3^Defined as a reduction in the size of the brain with a skull circumference less than three standard deviations below the mean.

^4^Infants/fetuses with multiple congenital heart defects affecting different subgroups are counted separately.

*Adjusted by using the quintiles of the propensity score incorporating maternal age, use of nicotine and alcohol, number of previous deliveries, number of previous spontaneous abortions, pregestational diabetes, folic acid intake preconception.

The first trimester maximum daily dose was higher for women with affected children than for women with children without major birth defects (median 1200 mg/day vs. 900 mg/day). Table 3 demonstrates the rising birth defect risks with higher daily doses, with the highest risk associated with exposure ≥ 1500 mg/day [OR_adj_ 5.41 (95% CI 2.32–12.66)]. By using a logistic regression model, an increase of the maximum dose by 100 mg/day raised the risk of major birth defects by 15% [OR 1.15 (95% CI 1.08–1.23)] (Fig. S4). Effects of VPA in antiepileptic polytherapy versus VPA monotherapy are summarized in Table 4 and Table S7. In comparison with the unexposed cohort, major birth defect risks were increased for both polytherapy [OR_adj_ 3.87 (95% CI 1.89–7.92)] and monotherapy [OR_adj_ 2.36 (95% CI 1.27–4.38)] (Table S7). However, for mono- and polytherapy (excluding teratogenic co-medication) with VPA dose < 1000 mg/day, the risk was not significantly increased (Table 4).

**Table 3:** Crude rates and results of logistic regression for major birth defects for VPA maximum dose categories < 700 mg/day, 700– < 1500 mg/day and ≥ 1500 mg/day in contrast to the comparison cohort.

	Major birth defects n (%)	OR (95% CI)	OR_adj_* (95% CI)
Comparison cohort	44/1306^1^ (3.4)	Reference	Reference
< 700 mg/day	5/135^2^ (3.7)	1.1 (0.43–2.83)	1.17 (0.44–3.13)
700– < 1500 mg/day	16/158^3^ (10.1)	3.23 (1.78–5.87)	3.43 (1.77–6.67)
≥ 1500 mg/day	9/60^4^ (15)	5.06 (2.34–10.92)	5.41 (2.32–12.66)

n = 40 liveborn infants had to be excluded from the analysis due to incomplete information of maximum VPA dose in first trimester including four major birth defects.

*CI* confidence interval, *ETOP* elective termination of pregnancy, *n* number of liveborn infants plus fetuses affected with major birth defects, *OR* odds ratio, *SAB* spontaneous abortion, *VPA* valproate.

^1^Including one SAB, five ETOP and one stillbirth.

^2^Including one ETOP and one stillbirth.

^3^Including one SAB and two ETOP.

^4^Including three ETOP.

*Adjusted by using the quintiles of the propensity score incorporating maternal age, use of nicotine and alcohol, number of previous deliveries, number of previous spontaneous abortions, pregestational diabetes, folic acid intake preconception.

**Table 4:** Crude rates and results of logistic regression for major birth defects for the different treatment regimes.

	Major birth defect n (%)	OR (95% CI)	OR_adj_* (95% CI)
Comparison cohort	44/1306^1^ (3.4)	Reference	Reference
Monotherapy < 1000 mg/day	5/124^2^ (4)	1.21 (0.47–3.1)	1.21 (0.45–3.22)
Monotherapy ≥ 1000 mg/day	15/115^3^ (13)	4.3 (2.31–8)	4.23 (2.09–8.57)
Polytherapy without teratogenic co-medication			
Polytherapy < 1000 mg/day	1/38^4^ (2.6)	0.78 (0.1–5.78)	0.76 (0.1–5.83)
Polytherapy ≥ 1000 mg/day	6/33 (18.2)	6.37 (2.5–16.22)	6.33 (2.38–16.82)
Polytherapy with teratogenic co-medication^6^	6/28^5^ (21.4)	7.82 (3.02–20.26)	7.71 (2.82–21.05)

The indicated dose refers to the maximum dose of VPA in the first trimester.

n = 17 liveborn infants with VPA monotherapy and seven with VPA polytherapy had to be excluded due to missing maximum dose in first trimester. When limiting polytherapy to lamotrigine and/or levetiracetam (n = 40), there was only one major birth defect with a maximum dose of VPA < 1000 mg/day.

*CI* confidence interval, *ETOP* elective termination of pregnancy, *IQR* interquartile range, *n* number of liveborn infants plus fetuses affected with major birth defects, *OR* odds ratio, *SAB* spontaneous abortion, *VPA* valproate.

^1^Including one SAB, five ETOP and one stillbirth.

^2^Including one ETOP.

^3^Including one SAB and three ETOP.

^4^Including one stillbirth.

^5^Including two ETOP.

^6^Including carbamazepine (n = 15), topiramate (n = 7), phenobarbital (n = 5), phenytoin (n = 3). Maximum dose of VPA in first trimester: median 1200 mg/day, IQR 900–1450 mg/day.

*Adjusted by using the quintiles of the propensity score incorporating maternal age, use of nicotine and alcohol, number of previous deliveries, number of previous spontaneous abortions, pregestational diabetes, folic acid intake preconception.

##### Treatment discontinuation

Effects of VPA discontinuation time during the first trimester on the risk of major birth defects are shown in Table 5 and Table S8. In contrast to the comparison cohort, the risk increase reached statistical significance when VPA was not discontinued during the first trimester of pregnancy [OR_adj_ 3.66 (95% CI 2.04–6.54)] (Table 5). However, women who discontinued VPA in the first trimester had a lower maximum dose of VPA compared to women who continued treatment of VPA throughout the first trimester [900 mg/day (IQR 500–1012.5 mg/day) vs. 1000 mg/day (IQR 600–1200 mg/day)]. Moreover, women with VPA monotherapy more frequently discontinued VPA in the first trimester than women on VPA polytherapy [39% (n = 98/251) vs. 31% (n = 29/95)], and only 22% (n = 57/257) discontinued within the epilepsy cohort. To address these confounders, several sensitivity analyses within the valproate exposed cohort were performed (Table S8). The risk of major birth defects was increased for continued treatment throughout first trimester in contrast to discontinuation until GW 13 [continued treatment 26/227 (11.5%) vs. discontinuation 8/150 (5.3%); OR 2.30 (95% CI 1.01–5.22)], and remained within the same range after adjusting for maximum dose, treatment indication, and mono- versus polytherapy, but did not reach statistical significance (Table S8).

**Table 5:** Crude rates and results of logistic regression for major birth defects grouped by early VPA discontinuation (≤ gestational week (GW) 5 + 0), moderate discontinuation (GW 5 + 1 − GW 7 + 0), late discontinuation (GW 7 + 1 − GW 12 + 6), and no VPA discontinuation in first trimester (GW ≥ 13 + 0) in contrast to the comparison cohort.

	Major birth defects n (%)	OR (95% CI)	OR_adj_* (95% CI)
Comparison cohort	44/1306^1^ (3.4)	Reference	Reference
Early discontinuation	2/48 (4.2)	1.25 (0.29–5.3)	1.24 (0.28–5.39)
Moderate discontinuation	3/64^2^ (4.7)	1.41 (0.43–4.67)	1.38 (0.4–4.78)
Late discontinuation	3/38 (7.9)	2.46 (0.73–8.3)	2.43 (0.69–8.49)
No discontinuation	26/227^3^ (11.5)	3.71 (2.23–6.16)	3.66 (2.04–6.54)

n = 16 liveborn infants had to be excluded from the analysis due to incomplete information of VPA exposure in first trimester.

*CI* confidence interval, *ETOP* elective termination of pregnancy, *n* number of liveborn infants plus fetuses affected with major birth defects, *OR* odds ratio, *SAB* spontaneous abortion, *VPA* valproate.

^1^Including one SAB, five ETOP and one stillbirth.

^2^Including two ETOP.

^3^Including one SAB, four ETOP and one stillbirth.

*Adjusted by using the quintiles of the propensity score incorporating maternal age, use of nicotine and alcohol, number of previous deliveries, number of previous spontaneous abortions, pregestational diabetes, folic acid intake preconception.

## Discussion

This is the first cohort study analyzing the effect of VPA discontinuation at different gestational stages during first trimester on the risk of SAB and congenital birth defects.

The overall risk of major birth defects determined by other studies varied between 6 and 15%^1–10^, which is in line with the rate of 8.7% in our study. Similar to other studies^2,6,9–11^ we found significantly increased risks for birth defects of the nervous system overall, the urinary system and particularly hypospadias in male infants. In addition, we observed increased ORs for spina bifida, the group of congenital heart defects_,_ oro-facial clefts, limb reduction defects and polydactyly, although they did not reach statistical significance possibly due to small numbers of events. A finding not yet reported by other working groups is the significantly increased rate of severe microcephaly [5/385 (1.3%) vs. 3/1299 (0.2%), OR_adj_ 6.65 (95% CI 1.17–37.68)]. At Embryotox, the diagnosis of microcephaly is based on reliable head circumference measures and not limited to formal coding of microcephaly, which may have been missed in clinical practice. It is worth mentioning, however, that severe microcephaly did not occur isolated in all cases, i.e. additional organ systems were affected (P0309 and P0600, Table S6). In addition, in four out of five infants affected with severe microcephaly, VPA monotherapy during the first trimester was reported (Table S6). All infants affected with severe microcephaly were born at term, while three infants were additionally small for gestational age (Table S6). To the best of our knowledge, only Jentink et al.^10^ reported an elevated but not significant risk for severe microcephaly [OR_adj_ 2.5 (95% CI 0.3–9.7)]. More recently, Blotíere et al.^2^ investigated the risk of severe microcephaly after monotherapy of ten different antiepileptic drugs, but could not find an increased risk for VPA. However, definition of severe microcephaly (both microcephaly < 3 SD without craniosynostosis (MedDRA Q02), but in addition with brain MRI by Blotiére et al.), time-point of diagnosis (at 24 months vs. at birth by Embryotox), and type of data (ICD-10 coding of health care data vs. measured head circumference at Embryotox) complicates a direct comparison.

It has been widely shown that the risk of birth defects is higher with increasing daily dose of VPA, although applied dose categories and associated risks vary from study to study^3–6,8,9,12^. Our study results are consistent with the risks and dose-categories applied by Tomson et al.^4,12^. However, we found slightly lower risks than their working group for VPA < 700 mg/day and ≥ 1500 mg/day^4,12^, which might be explained by differences in inclusion and exclusion criteria and length of follow-up. We omitted four cases with major birth defects from the analysis because of imprecise specification of the high dose (≥ 1000 mg/day). The effect of the maximum dose in first trimester was modelled linearly using a logistic regression. Based on this calculation, a risk increase of 15% was shown for each additional 100 mg/day VPA in the first trimester (Fig. S4). Risk calculation based on VPA plasma concentrations would have been advantageous but these data were not available.

AED polytherapy involving VPA has been associated with higher risks of birth defects than monotherapy^3,12–15^. We also found higher risk for VPA polytherapy than monotherapy (12.3% vs. 7.8%), which were both significantly increased compared to the non-exposed cohort. However, when assigning cases to either < 1000 mg/day or ≥ 1000 mg/day VPA, risks for mono- and polytherapy were comparable when teratogenic co-medication was excluded (Table 4). In contrast, birth defect risk was the highest when co-medication included teratogens, irrespective of the maximum VPA dose. The results of the present study support previous findings of Tomson et al.^12^ that VPA dose is of utmost importance in both mono- and polytherapy and that monotherapy in high doses might be more hazardous than polytherapy with lower VPA doses.

The most striking result of our study was the time-dependence of birth defect risk with a more than threefold risk increase compared to the non-exposed cohort when VPA treatment was not discontinued in the first trimester [11.5% vs. 3.4%, OR_adj_ 3.66 (95% CI 2.04–6.54)]. If VPA treatment was discontinued up to GW 5 + 0 the risk of major birth defects was only slightly increased (Table 5). The effect of continued treatment throughout the first trimester in contrast to treatment discontinuation remained significantly increased when comparing women in the VPA exposed cohort (Table S8, unadjusted OR). Several sensitivity analyses were performed to adjust for differences in maximum dose, mono- and polytherapy and treatment indication. The effects of continued treatment throughout the first trimester remained within the same range, but did not reach statistical significance possibly due to small event counts (Table S8). In addition, we were only able to adjust for one confounder at a time. Therefore, larger studies are needed to further investigate the effect of treatment discontinuation in the first trimester in a more complex setting. Moreover, we cannot rule out that treatment discontinuation is linked to other unmeasured risk factor such as severity of maternal disease, which might have biased our results.

Lower birth defect risk with early discontinuation during the first trimester was also observed with other teratogens such as vitamin K-antagonists^44^. However, in contrast to vitamin K-antagonists, no recommendations have been defined for AED treatment changes during pregnancy. Instead, a careful risk–benefit assessment is required in each individual patient, taking into account relapse risk in cases, where VPA has been shown to be the most effective treatment^26–33^.

In contrast to birth defects, there are only limited and conflicting data available regarding the risk of SAB associated with VPA. While some studies found increased risks for SAB^18–20,22^, others did not^1,9,21^. Our study neither indicates an overall increased rate of SAB [HR_adj_ 1.31 (95% CI 0.85–2.02)], nor dependence on treatment discontinuation. However, the high rate of ETOP as a competing event possibly results in an underestimation of SAB risk. A larger proportion of unwanted pregnancies, fear of VPA fetotoxicity, and severity of the underlying maternal disease may have contributed to the high ETOP rate among VPA exposed women. A limitation of our study is that information on treatment duration obtained through follow-up questionnaire was less complete in cases of pregnancy losses than in live births. Furthermore, estimation of gestational week in early pregnancy is mainly based on the first day of the last menstrual period (LMP), which might lead to slight inaccuracies in the estimation of pregnancy duration. A possible association between time of VPA discontinuation and SAB risk may therefore remain undetected.

Further strengths and limitations for pregnancy outcome studies using observational data were discussed in detail by Schaefer et al.^36^. Both cohorts of our study were collected following the Embryotox standardized data recording and processing protocol, making a substantial bias unlikely. Although the lost-to-follow-up rate is about 18%, Stegherr et al.^49^ showed that the difference in baseline covariates between response and non-response in the Embryotox cohort is unlikely to confound study results of drug toxicity. In addition, pregnant women registered at Embryotox may not be representative of pregnant women on VPA in Germany in general. For example, higher educational level of women who themselves or their treating physician seeking advice at the Embryotox center has already been demonstrated by Beck et al.^50^. On the other hand, in the study cohort analyzed here treatment indications for VPA are very similar for women of reproductive age in Germany as shown by claims data^51^.

## Conclusion

Our data suggest that treatment discontinuation of VPA in the first trimester may lower the risk of major birth defects. If VPA treatment during pregnancy cannot be avoided, the lowest possible dose should be prescribed in either mono- or polytherapy to achieve seizure control in patients with epilepsy.

### Supplementary Information

Supplementary Information.

## Data Availability

The data that support the findings of this study are available on request from the corresponding author. The data are not publicly available due to privacy or ethical restrictions.
