# Supplementary material for: Impact of maternal first trimester treatment regimen on the outcome of valproate exposed pregnancies: an observational Embryotox cohort study
Source: Sci Rep. 2024 Jan 5;14:674. doi: 10.1038/s41598-023-50669-1 (PMC10770162; doi:10.1038/s41598-023-50669-1)

**Supporting Information Scientific Reports**

**Impact of maternal first trimester treatment regimen on the outcome of valproate exposed pregnancies: an observational Embryotox cohort study**

Anne-Katrin Fietz*^1,2^, Marlies Onken^1^, Stephanie Padberg^1^, Christof Schaefer^1^, Katarina Dathe^1^

^1^Charité – Universitätsmedizin Berlin, corporate member of Freie Universität Berlin and Humboldt-Universität zu Berlin, Institute of Clinical Pharmacology and Toxicology, Embryotox Center of Clinical Teratology and Drug Safety in Pregnancy, Augustenburger Platz 1, 13353 Berlin, Germany

^2^Charité - Universitätsmedizin Berlin, corporate Member of Freie Universität Berlin and Humboldt-Universität zu Berlin, Institute of Biometry and Clinical Epidemiology, Charitéplatz 1, Berlin, Germany

***Correspondence:**

Anne-Katrin Fietz, Institute of Clinical Pharmacology and Toxicology, Embryotox Center of Clinical Teratology and Drug Safety in Pregnancy, Augustenburger Platz 1, 13353, Berlin, Germany. E-Mail: anne-katrin.fietz@charite.de

**Table S1:** Definitions of exclusion criteria, exposure, pregnancy outcomes and mono- and polytherapy.

|  | **Definition of exclusion criteria** |
| --- | --- |
| Exclusion criteria for study cohorts | Treatment with teratogens and fetotoxicants in the comparison cohort only: acenocoumarol, ACE-inhibitors and AT1-antagonists (in second and third trimester only), carbamazepine, lenalidomide, methotrexate, mycophenolate, phenobarbital, phenprocoumon, phenytoin, retinoids (acitretin, adapalen, isotretinoin, tazarotene and tretinoin), VPA.  Treatment of malignancies in both cohorts. MedDRA: malignant or unspecified tumors (SMQ 20000091), malignancy-related conditions (SMQ 20000092) ICD-10: C00-D09. |
| Gestational week (GW) | Calculation based on ultrasound measures in first trimester or by using the first day of the last menstrual period (LMP). |
| First trimester | Time between GW 2+0 and 12+6 after the first day of LMP. |
| SAB | Spontaneous pregnancy loss of a fetus <500 g or before GW 24+0 for unknown weight. |
| Preterm birth | Birth before GW 37+0. |
| Polytherapy | Parallel intake of antiepileptics (AEDs) (ATC-Code N03A) in addition to VPA for at least one day in first trimester. Due to their anticonvulsant properties, parallel exposure of benzodiazepines (ATC-Code N05B, N05C) was also considered as AED-polytherapy. |
| Monotherapy | VPA exposed pregnancies which were not exposed to any further AED during the first trimester. |
| Treatment changes | Treatment changes from VPA monotherapy to another AED were neither considered as mono- nor polytherapy and excluded from the analysis. |

AED: antiepileptics; GW: gestational week; LMP: first day of the last menstrual period; SAB: spontaneous abortion; VPA: valproate.

**Table S2:** Maternal characteristics and obstetric history of study cohorts.

|  | **VPA cohort**  **(n=484)** | **Comparison cohort**  **(n=1446)** |
| --- | --- | --- |
| **Maternal age*, n** | 478 | 1425 |
| Maternal age | 29 (24-33) (14-47) | 32 (28-35) (14-49) |
| **BMI*, n** | 327 | 1008 |
| BMI | 24.4 (21.7-28.3) (14.7-54.9) | 22.8 (20.7-25.7) (15.8-49.5) |
| **Educational level, n** | 188 | 744 |
| No leaving exam | 20 (10.6) | 7 (0.9) |
| 9 years exam | 36 (19.1) | 48 (6.5) |
| 10/11 years exam | 79 (42) | 227 (30.5) |
| Secondary school exam | 27 (14.4) | 179 (24.1) |
| Academic study | 26 (13.8) | 283 (38) |
| **Smoking*, n** | 456 | 1419 |
| No | 305 (66.9) | 1177 (82.9) |
| ≤ 5 cig/day | 31 (6.8) | 70 (4.9) |
| > 5 cig/day | 120 (26.3) | 172 (12.1) |
| **Alcohol*, n** | 453 | 1413 |
| No | 421 (92.9) | 1344 (95.1) |
| ≤ 1 drink/day | 16 (3.5) | 36 (2.5) |
| > 1 drink/day | 16 (3.5) | 33 (2.3) |
| **Social drugs, n** | 446 | 1387 |
| Yes | 12 (2.7) | 18 (1.3) |
| No | 434 (97.3) | 1369 (98.7) |
| **Pregnancy wanted, n** | 417 | 1185 |
| Yes | 327 (78.4) | 1075 (90.7) |
| Indifferent | 69 (16.5) | 87 (7.3) |
| No | 21 (5) | 23 (1.9) |
| **Previous pregnancies, n** | 479 | 1419 |
| 0 | 237 (49.5) | 626 (44.1) |
| 1 | 129 (26.9) | 387 (27.3) |
| 2 | 66 (13.8) | 214 (15.1) |
| 3 or more | 47 (9.8) | 192 (13.5) |
| **Previous deliveries*, n** | 480 | 1417 |
| 0 | 298 (62.1) | 749 (52.9) |
| 1 | 122 (25.4) | 399 (28.2) |
| 2 | 45 (9.4) | 202 (14.3) |
| 3 or more | 15 (3.1) | 67 (4.7) |
| **Previous SAB*, n** | 473 | 1413 |
| 0 | 407 (86) | 1180 (83.5) |
| 1 | 54 (11.4) | 163 (11.5) |
| 2 or more | 12 (2.5) | 70 (5) |
| **Previous ETOP, n** | 473 | 1413 |
| 0 | 413 (87.3) | 1316 (93.1) |
| 1 | 43 (9.1) | 77 (5.4) |
| 2 or more | 17 (3.6) | 20 (1.4) |
| **Previous children with major birth defect, n** | 474 | 1413 |
| 0 | 461 (97.3) | 1404 (99.4) |
| 1 | 13 (2.7) | 8 (0.6) |
| 2 or more | 0 (0) | 1 (0.1) |
| **Pregestational diabetes*, n** | 484 | 1446 |
| Yes | 4 (0.8) | 14 (1.0) |
| No | 480 (99.2) | 1432 (99.0) |
| **Folic acid intake preconception*, n** | 325 | 959 |
| Yes | 80 (24.6) | 404 (42.1) |
| No | 245 (75.4) | 555 (57.9) |
| **GW at first contact, n** | 483 | 1444 |
| GW at first contact | 7+6 (6+0-13+6) (1+5-39+4) | 8+6 (6+2-14+0) (2+0-41+4) |

*Baseline covariates used for adjustment. BMI: body mass index in kg/m^2^; ETOP: elective termination of pregnancy; GW: gestational week; n: number of pregnancies. SAB: spontaneous abortion; VPA: valproate. Median, interquartile range, and min/max are presented for maternal age, BMI and GW at first contact.

**Table S3:** Clinical indications and information on VPA treatment regime for each category.

| **Treatment indication** | **n (%)** | **Duration**  **in days median (IQR) (min-max)** | **Discontinuation^1^**  **n (%)** | **Maximum dose in mg/d**  **median (IQR)**  **(min-max)** | **Monotherapy**  **n (%)** | **Polytherapy**  **n (%)** |
| --- | --- | --- | --- | --- | --- | --- |
| Epilepsy | 333/484 (69%) | 250 (59-275)  (18-298) | 67/289  (23%) | 900 (600-1200)  (75-3000) | 174/295  (59%) | 121/295  (41%) |
| Bipolar disorder | 51/484  (11%) | 42 (35-54)  (1-281) | 43/47  (91%) | 1000 (600-1200)  (100-2000) | 36/42  (86%) | 6/42  (14%) |
| Psychotic disorder other than bipolar^2^ | 46/484  (10%) | 45 (36-91)  (3-291) | 26/43  (60%) | 1000 (600-1200)  (60-2000) | 36/40  (90%) | 4/40  (10%) |
| Affective disorder other than bipolar | 23/484  (5%) | 42 (36-66)  (22-287) | 18/21  (86%) | 950 (713-1025)  (50-1500) | 19/22  (86%) | 3/22  (14%) |
| Personality disorder | 17/484  (4%) | 41 (30-50)  (5-113) | 15/16  (94%) | 900 (450-1050)  (150-1400) | 13/15  (87%) | 2/15  (13%) |
| Migraine | 6/484  (1%) | 41 (37-113)  (31-253) | 4/6  (67%) | 700 (425-975)  (300-1000) | 6/6  (100%) | / |
| None of these indications | 8/484  (2%) | 43 (25-59)  (1-288) | 6/8  (75%) | 300 (300-800)  (150-1200) | 8/8  (100%) | / |

n: number of pregnancies; IQR: interquartile range; VPA: valproate.

^1^Detailed information on the course of VPA exposure in first trimester was available for 89% (n=430/484) of women.

^2^Including schizoaffective disorders.

**Table S4:** Additional substances of VPA polytherapy in first trimester. One AED in addition to VPA was used in 79% of the polytherapy pregnancies, and two or more in 21% (15% used two, 4% used three, 1% used four and 1% used five). Substances for women with more than one additional AED are counted separately.

| **Substances of polytherapy** | **n** |
| --- | --- |
| Lamotrigine | 64 |
| Levetiracetam | 22 |
| Carbamazepine | 18 |
| Clobazam | 12 |
| Topiramate | 11 |
| Lorazepam | 8 |
| Primidone | 8 |
| Diazepam | 6 |
| Phenobarbital | 5 |
| Clonazepam | 4 |
| Phenytoin | 4 |
| Lacosamide | 3 |
| Oxcarbazepine | 3 |
| Zonisamide | 2 |
| Barbexaclone | 1 |
| Benzobarbital | 1 |
| Bromazepam | 1 |
| Ethosuximide | 1 |
| Flunitrazepam | 1 |
| Gabapentin | 1 |
| Mesuximide | 1 |
| Temazepam | 1 |

n: number of pregnancies; VPA: valproate. VPA treatment was discontinued and replaced by another AED during the first trimester in 23 women (16 monotherapy, 7 polytherapy). Substances were lamotrigine (n=9), levetiracetam (n=4), carbamazepine (n=2), clobazam (n=2), benzodiazipine (n=1), diazepam (n=1), lamotrigine + levetiracetam (n=1), phenobarbital (n=1), phenytoin (n=1), clobazam + lamotrigine (n=1).

**Table S5:** Neonatal outcomes of study cohorts.

|  | **VPA** | **Comparison** | **Measure of association** | |
| --- | --- | --- | --- | --- |
| **Neonatal outcomes** | **n (%)** | **n (%)** | **OR**  **(95% CI)** | **OR_adj*_**  **(95% CI)** |
| Preterm birth^1^ | 43/371 (11.6) | 95/1245 (7.6) | 1.59 (1.08-2.32) | 1.47 (0.95-2.25) |
|  | **Median**  **(IQR)** | **Median**  **(IQR)** | **SDS diff**  **(95% CI)** | **SDS diff_adj§_ (95% CI)** |
| GW at birth | 39+1  (38+0-40+4) | 39+3  (38+0-40+3) |  |  |
| Birth weight in g | 3300  (2878-3626) | 3350  (3000-3700) | -0.16  (-0.28 to -0.03) | -0.12  (-0.24 to 0.01) |
| Head circumference in cm | 34.5  (33-36) | 35  (34-36) | -0.14  (-0.27 to -0.01) | -0.10  (-0.23 to 0.03) |

CI: confidence interval; GW: gestational week at birth; IQR: interquartile range; n: number of liveborn infants; OR: odds ratio; SDS diff: standard deviation score difference; VPA: valproate.

^1^Excluding multiple pregnancies.

*****adjusted by using the quintiles of the propensity score incorporating maternal age, use of nicotine and alcohol, number of previous deliveries, number of previous spontaneous abortions, pregestational diabetes, folic acid intake preconception.

§adjusted for maternal age, use of nicotine and alcohol, number of previous deliveries, number of previous spontaneous abortions, pregestational diabetes, folic acid intake preconception.

**Table S6:** Additional information for each study case with major birth defect in the first trimester exposed VPA cohort (n=34).

| **Study**  **no.** | **VPA exposure**  **from GW to GW (dose in mg/d)** | **Treatment indication** | **Age in yrs.** | **Completed GW at birth (outcome, sex), weight (W) and head (H) percentile (p)** | **Organ system affected by major birth defect** | **Major birth defects**  **(EUROCAT classification)** | **Co-exposure**  **(from GW to GW)^1^** |
| --- | --- | --- | --- | --- | --- | --- | --- |
| P0046 | 0 to 42  (1100-1500) | Epilepsy | 25 | 42 (LB, male)  W: 75.-90. p  H: 90.-97. p | Congenital heart defects | Ventricular septal defect (Q21.0) | - Antibiotics (14 to 16)  - Smoking (0 to 42) |
| P0204 | 0 to 27 (2500)  27 to 31  (2500-3600)  31 to 36 (3600) | Epilepsy | 37 | 36 (LB twin, male)  W: 3.-10. p  H: < 3. p | Congenital heart defects | Ventricular septal defect (Q21.0) | - Barbexaclone (0 to 36)  - Diazepam (27 to 36)  - Fenoterol (24, end unknown)  - Glucocorticoid (24, end unknown) |
| P0281 | 0 to 6+6 (1200)  7 to 40 (1000) | Epilepsy | 25 | 40 (LB, female)  W: 90.-97. p  H: NA | Oro-facial clefts | Cleft palate, unspecified (Q35.9) |  |
| P0309 | 4 to 4 (18000) | Suicide attempt | 18 | 37 (LB, female)  W: < 3. p  H: < 3. p (< 3 SD) | - Nervous system  - Digestive system  - Other anomalies/syndromes | - Severe microcephaly (Q02)  - Other specified congenital malformations of digestive system (Q45.8) (volvulus)  - Congenital absence of vertebra(e) (Q76.40) | Smoking (0 to 6+1, end unknown) |
| P0341 | 0 to 21+5 (1600)  21+6 to 38 (1500) | Epilepsy | 26 | 38 (LB, female)  W: 25.-50. p  H: 25.-50. p | - Oro-facial clefts  - Limb disorders | - Cleft palate (Q35)  - Accessory thumb(s) (Q69.1) | - Desogestrel (0 to 21)  - Folic acid (21+6, end unknown) |
| P0418 | 0 to 34+3 (2000, irregular intake) | Epilepsy | 33 | 34 (LB, male) W: 50.-75. p  H: 75.-90. p | Genital tract | Hypospadias, balanic (Q54.0) | Topiramate (0 to 34+3, irregular intake) |
| P0441 | 0 to 41 (1000) | Epilepsy | 26 | 41 (LB, female) W: 50.-75. p  H: 50.-75. p | Urinary system | Renal agenesis, unilateral (Q60.0) | - Budesonide (unknown trimester)  - Fluticasone & salmeterol (0 to 41, if required)  - Folic acid (0 to 41) |
| P0467 | 0 to 36+6 (1000) | Epilepsy | 20 | 36 (LB, female)  W: 75.-90. p  H: 75.-90. p | Congenital heart defects | Congenital malformation of cardiac septum, unspecified (Q21.9) | Folic acid (0 to 12+2, end unknown) |
| P0478 | 0 to 10 (1000) | Epilepsy | 32 | 38 (LB, male)  W: 75.-90. p  H: 50.-75. p | Urinary system | Atresia and stenosis of ureter (Q62.1) | Folic acid (0 to 6, end unknown) |
| P0487 | 0 to 15+1, end unknown (3000) | Epilepsy | 20 | 18 (ETOP, female) | - Congenital heart defects  - Oro-facial clefts  - Urinary system | - Ventricular septal defect (Q21.0)  - Transposition of great vessels (complete) (Q20.3)  - Cleft soft palate (Q35.3)  - Horseshoe kidney (Q63.10) | - Clobazam (5+5 to 15+1, end unknown)  - Folic acid (0 to 6, end unknown)  - Levetiracetam (1^st^ trimester)  - Phenobarbital (0 to 5+4) |
| P0511 | 0 to 40 (600) | Epilepsy | 30 | 40 (LB, male)  W: 25.-50. p  H: < 3. p (< 3 SD) | Nervous system | Severe microcephaly (Q02) | - Dexamethasone (0 to 9+4)  - Hydrocortisone (0 to 14) |
| P0600 | 0 to 21 (2100)  21+1 to 27+0 (600-2100)  27+1 to 41+4 (600) | Epilepsy | 28 | 41 (LB, male)  W: 10. p  H: < 3. p (< 3 SD) | - Nervous system  - Ear, face and neck | - Severe microcephaly (Q02)  - Congenital absence, atresia and stricture of auditory canal (external) (Q16.1) and congenital malformation of ear causing impairment of hearing, unspecified (Q16.9) | - Citalopram (0 to 27)  - Gabapentin (0 to 41+4)  - Levetiracetam (0 to 41+4)  - Lorazepam (if required)  - Metoclopramide (if required)  - Paracetamol (if required)  - Tolterodine (0 to 21, end unknown) |
| P0642 | 0 to 6+6 (1000)  7 to 22 (900) | Epilepsy | 26 | 22 (SAB, male) | - Respiratory  - Digestive system  - Genital tract  - Limb disorders | - Congenital malformation of lung, unspecified (Q33.9)  - Congenital absence, atresia, stenosis of anus without fistula (Q42.3)  - Hypospadias, penoscrotal (Q54.2)  - Longitudinal reduction defect of radius (Q71.4) | Folic acid (6 to 22) |
| P0643 | 0 to 40 (1000) | Epilepsy | 37 | 40 (LB, male)  W: 10.-25. p  H: 25.-50. p | Congenital heart defects | - Coarctation of aorta (Q25.1) | - Carbamazepine (0 to 40)  - Folic acid (5 to 8, end unknown)  - Topiramate (0 to 40) |
| P0777 | 0 to 5+1 (600) | Psychotic disorder | 23 | 39 (LB, male)  W: 3.-10. p  H: < 3. p (< 3 SD) | Nervous system | Severe microcephaly (Q02) | - Escitalopram (0 to 5+1)  - Folic acid (5 to 7+1, end unknown)  - Quetiapine (0 to 5+1)  - Trimipramine (0 to 5+1) |
| P0788 | 0 to 30 (1250)  30+1 to 38+1 (1500) | Epilepsy | 37 | 38 (LB, female)  W: < 3. p  H: 50.-75. p | Other anomalies/syndromes | Craniosynostosis (Q75.0) |  |
| P0791 | 0 to 37+5 (1200) | Epilepsy | 30 | 37 (LB, male)  W: 3.-10. p  H: 25.-50. p | Genital tract | Hypospadias, balanic (Q54.0) | - Carbamazepine (0 to 37+5)  - Folic acid (7+1, end unknown)  - Smoking (0 to 37+5) |
| P0945 | 0 to 31+1 (1350)  31+2 to 32+3 (1650)  32+4 to 38+2 (1800)  38+3 to 38+5 (1950)  38+6 to 39+3 (2100) | Epilepsy | 24 | 39 (LB, male)  W: 50.-75. p  H: 25.-50. p | - Congenital heart defects  - Urinary system  - Genital tract | - Atrial septal defect, ASD (Q21.1)  - Horseshoe kidney (Q63.10)  - Hypospadias, balanic (Q54.0) | - Betamethasone (3^rd^ trimester)  - Clonazepam (31+2 to 38+6)  - Folic acid (0 to 39+3) |
| P1002 | 0 to 14+2 (3000) | Epilepsy | 28 | 20 (ETOP, NA) | - Nervous system  - Oro-facial clefts  - Limb disorders | - Lumbar spina bifida without hydrocephalus (Q05.7)  - Cleft palate with cleft lip (Q37)  - Longitudinal reduction defect of radius (Q71.4) | - Clobazam (0 to 20+4)  - Lacosamide (16 to 19+2)  - Lamotrigine (0 to 20+4)  - Levetiracetam (0 to 20+4)  - Levothyroxine (0 to 20+4)  - Lorazepam (0 to 19+4, if required)  - Phenobarbital (0 to 20+4)  - Phenytoin (0 to 20+4) |
| P1033 | 0 to 28+2 (1200) | Schizoaffective disorder | 33 | 38 (LB, male)  W: 10.-25. p  H: 75. p | Genital tract | Hypospadias, balanic (Q54.0) | - Amitriptylin (30 to 37+4)  - Clozapine (0 to 38+3)  - Citalopram (37+3 to 38+3)  - Homeopathic drug (34+5 to 38+3, if required)  - Lactulose (35 to 35, if required)  - Lithium (28 to 29, start and end unknown)  - Magaldrate (35 to 38+2, if required)  - Quetiapine (0 to 28+2)  - Smoking (0 to 38+3)  - Valeriana officinalis root (34+4 to 38) |
| P1163 | 0 to 22+2 (500) | Epilepsy | 17 | 22 (ETOP, male) | Congenital heart defects | Atrial septal defect, ASD (Q21.1) | - Copper IUD (0 to 21+6)  - Glucocorticoid (19+6 to 20+6) |
| P1224 | 0 to 3+1 (600) | Bipolar disorder | 23 | 41 (LB, male)  W: 25.-50. p  H: 50.-75. p | Oro-facial clefts | Unspecified cleft palate with unilateral cleft lip (Q37.9) | - Antibiotics (1^st^ trimester)  - Clotrimazole (unknown trimester)  -Folic acid (4+5 to 13, end unknown)  - Quetiapine (0 to 41+4) |
| P1236 | 0 to 6+2 (1000)  6+3 to 8+3 (450)  8+4 to 10+4 (300)  10+5 to 12+5 (150) | Epilepsy | 34 | 39 (LB, male)  W: 25.-50. p  H: 25.-50. p | Urinary system | Double urethra (Q64.72) | - Folic acid (2+3 to 7+1)  - Lamotrigine (12+5 to 39) |
| P1299 | 0 to 40  (600-800) | Epilepsy | 30 | 40 (LB, female)  W: 3.-10. p  H: < 3. p (< 3 SD) | Nervous system | Severe microcephaly (Q02) | - Acetylsalicylic acid (20 to 36)  - Folic acid (0 to 6+3, end unknown)  - Levothyroxine (0 to 40) |
| P1402 | 0 to 9+6 (1200) | Epilepsy | 23 | 39 (LB, female)  W: 3.-10. p  H: 10.-25. p | - Congenital heart defects  - Urinary system | - Ventricular septal defect (Q21.0)  - Multicystic dysplastic kidney, unilateral (Q61.40) | - Carbamazepine (21+1 to 39+2)  - Clobazam (11 to 39+2)  - Folic acid (11 to 21, end unknown)  - Lamotrigine (11 to 19+1)  - Levothyroxin (0 to 39+2)  - Topiramate (0 to 39+2) |
| P1403 | 0 to 38+4 (2000) | Epilepsy | 27 | 38 (LB, male)  W: 25.-50. p  H: 3.-10. p | Limb disorders | Fused toes (Q70.2) | - Amphetamine (0 to 10, irregular intake)  - Primidone (0 to 13+6, end unknown)  - Smoking (unknown trimester) |
| P1404 | 0 to 30 (600) | Epilepsy | 23 | 30 (Stillborn, NA) | Congenital heart defects | Congenital pulmonary valve stenosis (Q22.1) | - Folic acid (5 to 8)  - Lamotrigine (4+5 to 6+5)  - Smoking (0 to 9, end unknown) |
| P1444 | 0 to 38+4 (1000-1500) | Epilepsy | 24 | 38 (LB, male)  W: 25.-50. p  H: 25.-50. p | Genital tract | Hypospadias, balanic (Q54.0) | - Primidone (0 to 38+4)  - Smoking (0 to 32+4, end unknown) |
| P1453 | 0 to 6 (1000) | Psychotic disorder | 26 | 22 (ETOP, female) | Nervous system | Lumbar spina bifida with hydrocephalus (Q05.2) and Arnold-Chiari syndrome (Q07.0) | - Folic acid (6+2 to 6+3, end unknown)  - Quetiapine (0 to 7+5, end unknown) |
| P1651 | 0 to 6 (2000)  6+1 to 20+5, end unknown (1000) | Epilepsy | 21 | 22 (ETOP, NA) | Nervous systems | Spina bifida, unspecified (Q05.9) | - Contraceptives (1^st^ trimester) |
| P1728 | 0 to 5+5 (1400) | Borderline personality disorder | 26 | 16 (ETOP, NA) | Nervous system | Lumbar spina bifida without hydrocephalus (Q05.7) | - Agomelatine (early 1^st^ trimester)  - Cannabis (0 to 5+5)  - Chlorprothixene (0 to 5+5)  - Citalopram (10 to 16+5)  - Doxepin (0 to 5+5)  - Omeprazole (0 to 7+2, end unknown)  - Pantoprazole (unknown trimester)  - Promethazine (6 to 16+5)  - Smoking (0 to 16+5, end unknown)  - Amphetamines (0 to 5+5) |
| P1841 | 0 to 38+5 (1000) | Epilepsy | 26 | 38 (LB, male)  W: 3.-10. p  H: NA | Limb disorders | Accessory finger(s) (Q69.0) | Folic acid (unknown trimester) |
| P1899 | 0 to 37+2 (1200-1500) | Epilepsy | 19 | 37 (LB twin, male)  Neonate 1:  W: 25.-50. p  H: 25.-50. p  Neonate 2:  W: 90.-97. p  H: 50.-75. p | Nenonate 1:  - Nervous system  - Genital tract  Neonate 2:  Nervous system | Neonate 1:  - Agyria or lissencephaly (Q04.33)  - Hypospadias, balanic (Q54.0)  Neonate 2:  Agyria or lissencephaly (Q04.33) | - Folic acid (unknown trimester)  - Oxcarbazepine (0 to 37+2) |

^1^Folic acid intake was either unknown or not applied if not listed

ETOP: elective termination of pregnancy; GW: gestational week; H: child head circumference at birth; LB: live birth; NA: not available; P: percentile according to Voigt et al. 2014 for singletons and twins; SAB: spontaneous abortion; SD: standard deviation; VPA: valproate; W: child weight at birth.

**Table S7:** Crude rates and results of logistic regression for major birth defects for VPA mono- and polytherapy in first trimester of pregnancy in contrast to the comparison cohort.

|  | **Major birth defects**  **n (%)** | **OR**  **(95% CI)** | **OR_adj*_**  **(95% CI)** |
| --- | --- | --- | --- |
| Comparison cohort | 44/1306^1^ (3.4) | Reference | Reference |
| Monotherapy | 20/256^2^ (7.8) | 2.43 (1.41-4.2) | 2.36 (1.27-4.38) |
| Polytherapy | 13/106^3^ (12.3) | 4.01 (2.09-7.71) | 3.87 (1.89-7.92) |

CI: confidence interval; ETOP: elective termination of pregnancy; OR: odds ratio; n: number of liveborn infants plus fetuses affected with major birth defects; SAB: spontaneous abortion; VPA: valproate.

^1^Including one SAB, five ETOP and one stillbirth.

^2^Including one SAB and four ETOP.

^3^Including two ETOP and one stillbirth.

*****adjusted by using the quintiles of the propensity score incorporating maternal age, use of nicotine and alcohol, number of previous deliveries, number of previous spontaneous abortions, pregestational diabetes, folic acid intake preconception.

**Table S8:** Effect of continued VPA treatment throughout first trimester (exposure of VPA ≥ GW 13) versus treatment discontinuation in first trimester (exposure of VPA < GW 13) unadjusted and adjusted for the maximum dose in first trimester, mono- and polytherapy and subgroup analysis for treatment indication epilepsy.

| **Effect for continued VPA treatment versus treatment discontinuation in first trimester** |  | **OR (95% CI)** |
| --- | --- | --- |
| Unadjusted |  | 2.30 (1.01-5.22) |
| Dose adjusted (< and ≥ 1000 mg/d) |  | 2.08 (0.90 -4.82) |
| Mono/polytherapy adjusted |  | 2.18 (0.92 -5.19) |
| Mono/polytherapy and dose adjusted (< and ≥ 1000 mg/d) |  | 2.04 (0.84 -4.96) |
| Unadjusted within epilepsy |  | 2.49 (0.72 -8.55) |
| Dose adjusted (< and ≥ 1000 mg/d) within epilepsy |  | 2.01 (0.56-7.18) |

CI: confidence interval; GW: gestational week; OR: odds ratio; VPA: valproate.

**Figure S1:** Flowchart for number of requests (n) on VPA exposure following the study criteria.


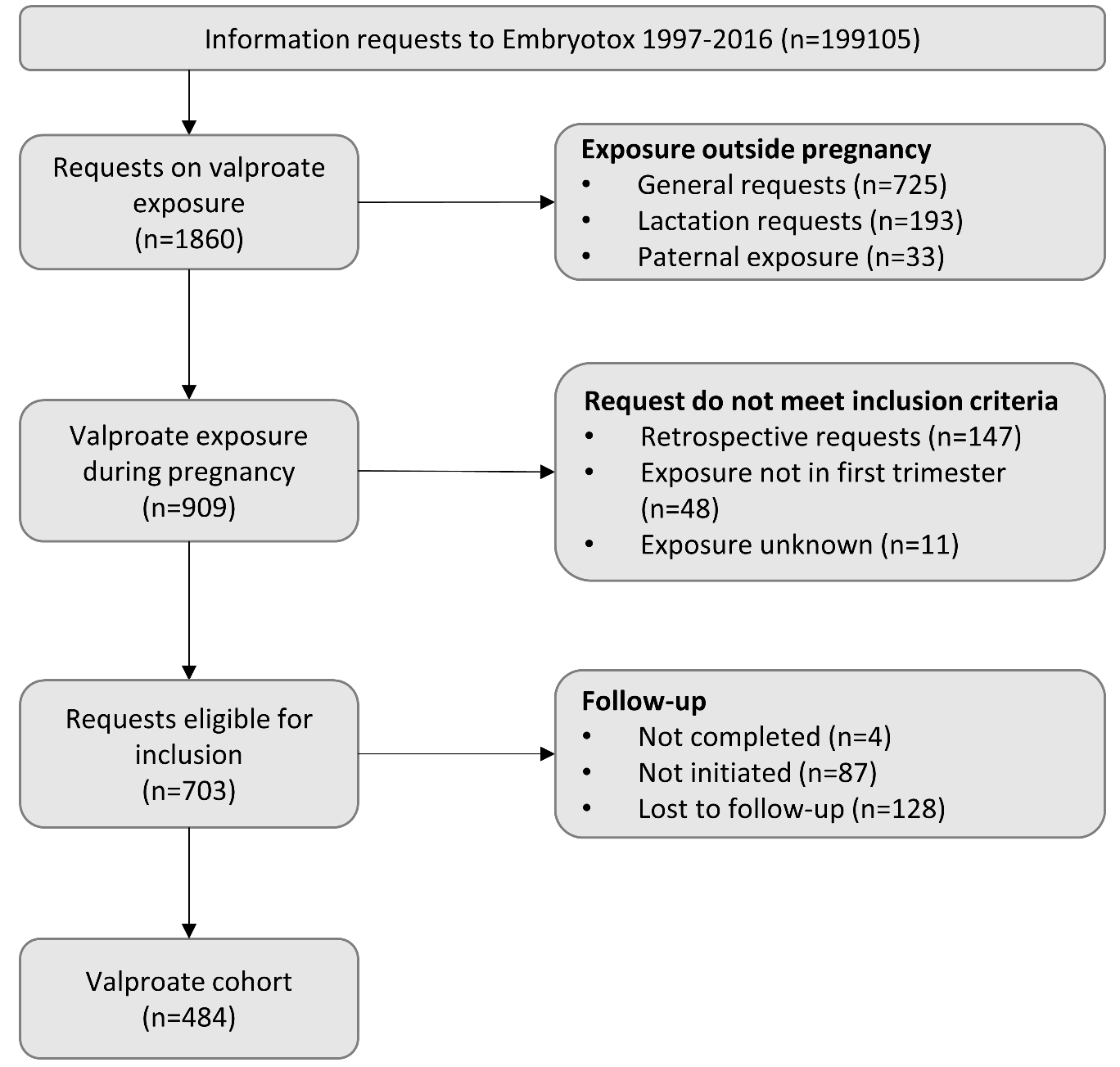


**Figure S2:** Number of pregnancies with VPA discontinuation in pregnancy (n=201) by gestational week.

**Figure S3:** Stacked cumulative incidences in the study cohorts for the pregnancy outcomes live birth, spontaneous abortion, elective termination of pregnancy (ETOP) and stillbirth alongside with the number of pregnancies at risk for each gestational day (dotted line). For multiple pregnancies with different pregnancy outcomes, each outcome was considered, while for multiple pregnancies with identical outcomes, only one outcome was considered.

**Figure S4:** Logistic regression lines for the risk of maximum dose in first trimester of valproate (VPA) in 100 mg/d units on major birth defects, with the following regression equation: logit risk = -3.95451 + 0.14270 dose, p < 0.0001. Black dots represent children without major birth defects (bottom line) and with major birth defects (top line) with the corresponding maximum dose of VPA in first trimester.


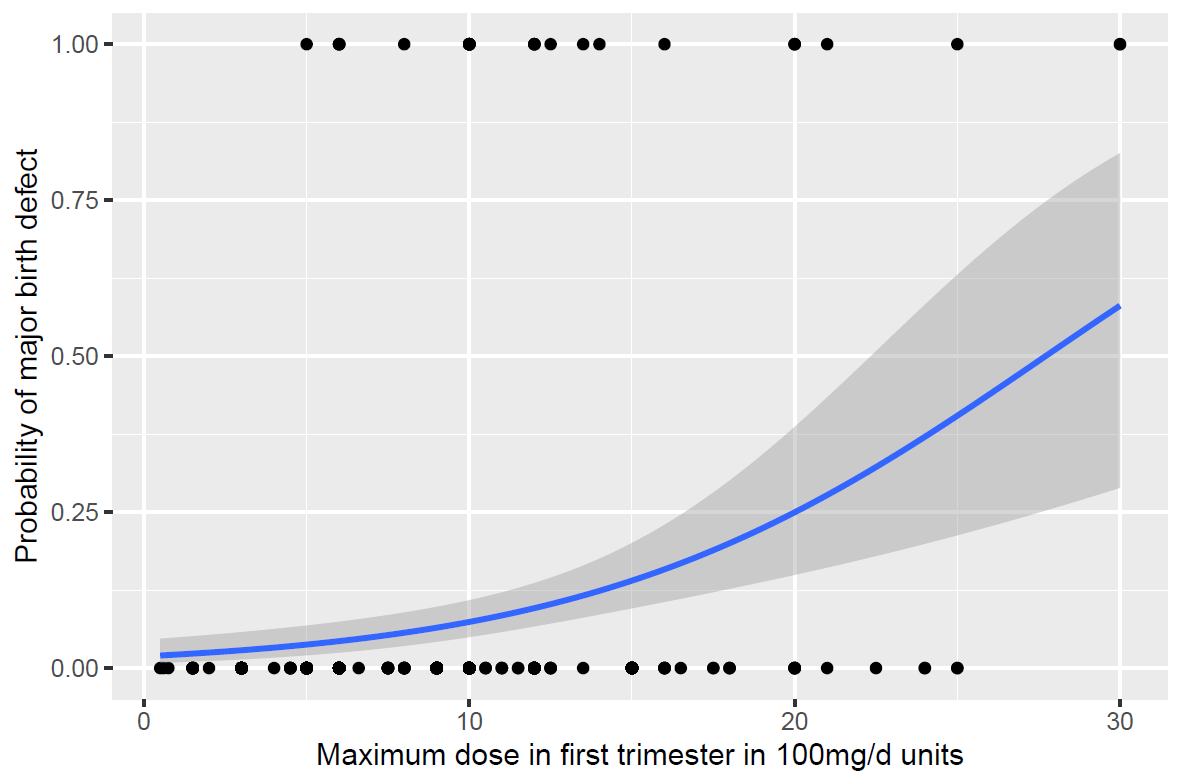

Supplement: Supplementary file 1 — Supplementary Information. [file 41598_2023_50669_MOESM1_ESM.docx]
